# Photoregulation of lipid metabolism in *Begonia*: synergistic effects of spectral composition and intensity on stress adaptation

**DOI:** 10.3389/fpls.2025.1746063

**Published:** 2026-01-16

**Authors:** Zhongke Wang, Hongbo Fu, Hongzheng Tao, Peina Ju, Hao Wei, Weichang Gong, Li Zhuang

**Affiliations:** 1 College of Life Science, Shihezi University, Shihezi, China; 2 College of Biological and Agricultural Sciences, Honghe University, Mengzi, China; 3 Institute of Advanced Agricultural Technology, Qilu Normal University, Jinan, China; 4 School of Life Science, Qufu Normal University, Qufu, China

**Keywords:** antioxidant response, *Begonia*, interactive effects, light, lipid metabolites

## Abstract

Light quality and intensity are pivotal environmental cues regulating plant lipid metabolism, yet their combined effects in shade-adapted species remain poorly understood. This study investigates how white (W), red (R), and blue (B) light at intensities of 5, 20, and 50 μmol·m^-2^·s^-1^ modulate lipid remodeling in *Begonia* ‘Black Velvet’. Using integrated lipidomics and transcriptomics, we detected 492 lipid metabolites, with steroids (23.17%) and isoprenes (21.95%) predominating. Principal component analysis revealed that light intensity exerted stronger discriminatory power than spectral quality on global lipid profiles. We annotated 443 differential lipid metabolites (DLMs), including 25 influenced primarily by light quality (e.g., pregnanetriol under blue light) and 23 by light intensity (e.g., glyceroglycolipids peaking at 20 μmol·m^-2^·s^-1^). Notably, 409 DLMs showed an interaction between the two factors. Physiological profiling linked key lipids (e.g., chabrolosteroid E, culobophylin C) to antioxidant enzymes (POD, SOD) and oxidative stress markers (MDA). Transcriptomics highlighted regulatory roles for *ERG* genes in steroid biosynthesis and *accD*/*FabF* in fatty acid pathways. Our findings demonstrate an association where *Begonia* shifts its lipid metabolism in response to light, correlating with adjustments in energy storage, and stress resilience, potentially involving optimization of membrane properties.

## 1 Introduction

Light is a fundamental environmental factor regulating plant growth, development, and metabolic adaptation. Both light quality (spectral composition) and intensity (photon flux density) act as key signals that influence photosynthetic efficiency, photomorphogenesis, and stress acclimation (Eghbal et al., 2024; Wu et al., 2025). Red and blue wavelengths are particularly influential, mediating processes such as photoperiodic flowering, stomatal movement, chloroplast development, and circadian regulation through dedicated photoreceptor systems (Chibani et al., 2025; Trivellini et al., 2023). Meanwhile, light intensity is a key factor influencing the rate of carbon assimilation and energy supply, with both excess and deficient light triggering distinct physiological and molecular responses that impact membrane integrity, redox homeostasis, and long-term fitness (Ruban and Belgio, 2014; Tang et al., 2022).

Lipid metabolites serve as essential structural components of cellular membranes, key reservoirs of energy, and dynamic signaling molecules involved in plant environmental sensing and adaptation (Gong et al., 2024; Rodríguez et al., 2025). In sun-adapted species such as *Arabidopsis thaliana* and *Lactuca sativa*, light-driven lipid remodeling has been well documented: blue light typically upregulates the synthesis of polyunsaturated fatty acids (PUFAs) through photoreceptor CRY1-mediated activation of fatty acid desaturase genes, thereby enhancing membrane fluidity and cold tolerance (Ma et al., 2016). In contrast, red light promotes the accumulation of specific phospholipids such as phosphatidylglycerol (PG), which supports the assembly and stability of photosystem II complexes (Zhang and Aro, 2002; Sawatdee et al., 2023). These responses are largely optimized for performance under moderate to high light. In contrast, plants adapted to persistent low-light understory environments, such as shade-tolerant species, are likely to employ divergent lipid metabolic strategies that prioritize membrane stability, efficient energy storage, and mitigation of intermittent light stress, rather than maximizing photosynthetic output (Maryam et al., 2025). However, these responses have predominantly been characterized under moderate to high light intensities, and much less is known about how plants adapted to persistent low-light conditions adjust their lipid metabolism under variable spectral environments.

*Begonia* represents one of the largest genera of shade-adapted understory plants, with species that have evolved under the canopy of tropical and subtropical forests. Compared to sun-adapted model plants, *Begonia* exhibits a suite of distinct morphological and physiological traits that optimize light capture and utilization under limited illumination, including thin laminar leaves with large surface areas, reduced stomatal density, and chloroplasts adapted to low-light conditions (Xiong et al., 2023; Feng et al., 2022). These adaptations suggest that *Begonia* may employ unique lipid metabolic strategies to maintain membrane functionality, manage energy reserves, and mitigate oxidative stress in light environments that fluctuate in both spectrum and intensity. Despite its ecological significance and horticultural importance, the interactive effects of light quality and intensity on lipid metabolism in *Begonia*, and the potential synergy between these two factors, remain completely unexplored at the omics level. Addressing this gap is crucial not only for understanding the fundamental ecological physiology of shade-adapted plants but also for revealing potential novel lipid-based adaptation mechanisms that differ from the established paradigms in sun-adapted models.

In this study, we hypothesize that in the shade-adapted *Begonia*, light intensity exerts a stronger influence than spectral quality on global lipid remodeling, and that this species exhibits distinct lipid metabolic responses compared to sun-adapted models, particularly through enhanced accumulation of sterols and unsaturated fatty acids to maintain membrane stability and energy reserves under variable light regimes. To test this hypothesis, we subjected *Begonia* ‘Black Velvet’, a popular ornamental cultivar with pronounced shade tolerance to three light qualities (white, red, blue) across three intensities (5, 20, 50 μmol·m^-2^·s^-1^), to represent a range of understory to moderate light conditions. Using an integrated multi-omics approach combining lipidomics, transcriptomics, and physiological profiling, we aimed to: (1) systematically identify lipid metabolites and pathways responsive to light quality and intensity, (2) evaluate how lipid changes correlate with antioxidant enzyme activities and oxidative stress markers, and (3) uncover key genes underlying light-induced lipid metabolic reprogramming.

This study provides novel insights into the photoregulation of lipid metabolism in a shade-adapted plant system, highlighting species-specific adaptive strategies that differ from those of sun-adapted models. Our findings not only advance the fundamental understanding of plant environmental adaptation but also offer practical guidelines for optimizing light regimes in the cultivation of *Begonia* and other shade-tolerant ornamental species.

## 2 Materials and methods

### 2.1 Plant materials and treatments

The experiment was conducted from August 2024 to February 2025 in a light-quality controlled chamber at the School of Life Sciences, Qufu Normal University. Test materials consisted of perennial *Begonia* ‘Black Velvet’ seedlings purchased from the Weifang Flower Market. All seedlings originated from the same batch and supplier. Following, a one-month acclimatization period in the Qufu Normal University laboratory, individuals exhibiting consistent size and growth vigor were selected for experimental treatments.

Specifically, treatments were administered within plant growth chambers maintained at 22°C, 85-90% relative humidity, and a 6-h light/18-h dark cycle. The experimental design incorporated two environmental factors: light intensity and light quality. Light treatments were provided by LED modules (Jiada Lighting Co., Ltd., China; models: White light: W5730-12V, Red light: R5730-12V, Blue light: B5730-12V). The spectral power distribution of each LED type at the three intensity levels is provided in **Supplementary File 1 Table 1**. Each factor had three levels: light intensities of 5, 20, and 50 μmol·m^-2^·s^-1^; and light qualities of white, red, and blue light. Consequently, this resulted in nine distinct treatment combinations including 5 μmol·m^-2^·s^-1^ white light (W5), 20 μmol·m^-2^·s^-1^ white light (W20), 50 μmol·m^-2^·s^-1^ white light (W50), 5 μmol·m^-2^·s^-1^ red light (R5), 20 μmol·m^-2^·s^-1^ red light (R20), 50 μmol·m^-2^·s^-1^ red light (R50), 5 μmol·m^-2^·s^-1^ blue light (B5), 20 μmol·m^-2^·s^-1^ blue light (B20), 50 μmol·m^-2^·s^-1^ blue light (B50). For each treatment, nine pots of plants were utilized, with three pots constituting one replicate, thereby providing three biological replicates per treatment. All plant materials were grown under these controlled conditions for 14 days. Subsequently, newly developed leaves were harvested as experimental samples, flash-frozen in liquid nitrogen, and stored for subsequent analyses.

### 2.2 Analysis of lipid metabolites

#### 2.2.1 Metabolites extraction

Following vacuum freeze-drying, approximately 50 mg of each sample was weighed and transferred into an extraction tube. Subsequently, 1000 μL of extraction solvent (methanol/acetonitrile/water, 1:2:1, v/v/v) was added, followed by vortex mixing for 30 seconds. Steel beads were then added, and homogenization was performed using a ball mill at 45 Hz for 10 min. Following this, the samples were sonicated for 10 min with ice-water bath cooling and incubated at -20 °C for 1 hour. Thereafter, the samples were centrifuged at 4 °C and 12,000 × *g* for 15 min. Finally, 300 μL of the supernatant was carefully transferred, filtered through a 0.22-μm organic filter membrane into a 2 mL autosampler vial, and 10 μL aliquots from each sample were pooled to generate a quality control (QC) sample for LC-MS analysis.

#### 2.2.2 LC-MS/MS analysis

Sample extracts were analyzed using an UPLC-ESI-MS/MS system (UPLC: Waters Acquity I-Class PLUS; MS: Applied Biosystems QTRAP 6500^+^). The analytical conditions were as follows. UPLC conditions: Column, Waters HSS-T3 (1.8 µm, 2.1 mm × 100 mm); mobile phase, solvent A (pure water with 0.1% formic acid and 5 mM ammonium acetate) and solvent B (acetonitrile with 0.1% formic acid). The gradient program employed initial conditions of 98% A and 2% B, maintained for 1.5 min. Subsequently, a linear gradient to 50% A and 50% B was applied within 5.0 min, followed by a linear gradient to 2% A and 98% B within 9.0 min; this composition (2% A, 98% B) was held for 1.0 min. Finally, the composition was readjusted to 98% A and 2% B within 1.0 min and maintained for 3.0 min. The flow rate was 0.35 mL/min, the column oven temperature was 50 °C, and the injection volume was 4 μL. The column effluent was directed to an ESI-triple quadrupole-linear ion trap (QTRAP)-MS.

ESI source parameters were set as follows: source temperature, 550 °C; ion spray voltage, 5500 V (positive ion mode) or -4500 V (negative ion mode); ion source gas I, gas II, and curtain gas, 50, 55, and 35 psi, respectively; collision-activated dissociation, medium. Instrument tuning and mass calibration were performed using 10 μmol/L and 100 μmol/L polypropylene glycol solutions in QQQ and LIT modes, respectively. For QQQ scans, Multiple Reaction Monitoring (MRM) experiments were acquired with collision gas (nitrogen) set to medium. Declustering potential and collision energy for individual MRM transitions were optimized; specific MRM transitions were monitored during each chromatographic period according to the elution profile of metabolites.

#### 2.2.3 Data analysis

Based on the in-house GB-PLANT database, metabolite detected and annotated was performed using MS/MS spectra. During analysis, isotopic signals, redundant signals from K^+^, Na^+^, and NH_4_^+^ ions, as well as redundant fragment ions originating from higher molecular weight compounds were excluded. Subsequently, metabolite quantification was conducted using MRM mode on a triple-quadrupole mass spectromete. In MRM mode, the first quadrupole filters precursor ions (parent ions) of target metabolites, thereby preliminarily eliminating interferences from ions of other molecular weights. These precursor ions are then fragmented into multiple product ions via collision-induced dissociation in the collision cell. A specific characteristic product ion is subsequently selected by the third quadrupole, which further excludes non-target ion interferences, thereby enhancing quantification accuracy and reproducibility.

Following acquisition of metabolomic data across all samples, peak area integration was performed for all metabolites, with subsequent cross-sample correction applied to peaks corresponding to identical metabolites. After normalizing the original peak area information against the total peak area, downstream analyses were conducted. Detected metabolites were annotated using the KEGG, HMDB, and Lipidmaps databases to obtain classification and pathway information. Furthermore, fold changes (FC) were calculated based on grouping information, while Student’s t-tests were performed to determine the significance (*p*-value) of differential abundance for each metabolite. Orthogonal Partial Least Squares-Discriminant Analysis (OPLS-DA) modeling was implemented using the R package ropls, and model reliability was validated through 200 permutation tests. Variable Importance in Projection (VIP) values were calculated via multiple cross-validation. Differential lipid metabolites were screened by integrating three criteria: fold change (FC > 1), significance level (*p*-value < 0.05), and OPLS-DA VIP value (VIP > 1). Finally, significantly enriched KEGG pathways among differential lipid metabolites were identified using the hypergeometric distribution test.

### 2.3 RNA-sequencing

#### 2.3.1 RNA quality assessment

Transcriptome sequencing was performed by Biomarker Technologies Co., Ltd (Beijing, China). To ensure sample qualification, the company employed advanced molecular biology instrumentation for comprehensive assessment of total RNA purity, concentration, and integrity, implementing stringent quality control throughout the process. Specifically, RNA purity and concentration were determined using NanoDrop 2000 spectrophotometry, which calculated A260/A280 and A260/A230 ratios, with concentration expressed in ng/μL. Furthermore, RNA integrity was precisely evaluated using either the Agilent 2100 Bioanalyzer or LabChip GX system, generating RNA Integrity Number values.

#### 2.3.2 Library construction, quality control and sequencing

Following successful RNA qualification, cDNA libraries were prepared through this workflow: Eukaryotic mRNA was enriched using Oligo(dT) magnetic beads and randomly fragmented via fragmentation buffer under elevated temperature. Using fragmented mRNA as template, first-strand cDNA synthesis was performed, followed by second-strand cDNA synthesis and purification. The purified double-stranded cDNA underwent end repair, adenylation, and adapter ligation, after which size selection was conducted using AMPure XP beads. Finally, libraries were amplified through PCR enrichment. Subsequently, library quality assessment employed a three-tiered approach: Preliminary quantification using Qubit 3.0 fluorometer confirmed concentrations ≥ 1 ng/μL. Insert size distribution analysis via Qsep400 high-throughput electrophoresis verified fragment integrity. Absolute quantification of effective library concentration (> 2 nM) was achieved through qPCR. Following successful QC, PE150 paired-end sequencing was performed on a high-throughput platform by Biomarker Technologies Co., Ltd (Beijing, China).

#### 2.3.3 Gene expression analysis

Paired-end reads were aligned to the Unigene reference using Bowtie. Subsequently, expression levels were quantified via RSEM based on alignment results, with abundance expressed as FPKM (Fragments Per Kilobase of transcript per Million mapped reads). For differential expression analysis, DESeq2 identified differentially expressed genes (DEGs) using raw count data across samples. Specifically, DEGs were selected under stringent thresholds: absolute Fold Change ≥ 2 and False Discovery Rate (FDR) < 0.01, where FDR was calculated using Benjamini-Hochberg correction to control type I errors. Finally, log_2_-transformed fold changes (log_2_FC) were used for visualization, with larger absolute log_2_FC values and smaller FDR values indicating higher statistical significance of differential expression between groups.

#### 2.3.4 Functional annotation analysis

Unigene sequences were aligned against multiple databases, including NR, Swiss-Prot, COG (Clusters of Orthologous Groups), KOG (euKaryotic Orthologous Groups), eggNOG v4.5, and KEGG using DIAMOND software. Subsequently, KOBAS assigned KEGG Orthology (KO) terms. In parallel, Gene Ontology (GO) annotations were obtained through InterProScan via integration of InterPro databases. Following amino acid sequence prediction, Pfam domain annotations were generated using HMMER hmmscan alignment.

### 2.4 Antioxidant enzyme activities

#### 2.4.1 Superoxide dismutase activity

Leaf samples (0.4 g) from different treatments were homogenized thoroughly in an ice bath with 4 mL of enzyme extraction buffer (50 mmol/L PBS, pH 7.8, containing 0.1 mmol/L EDTA), using 2 mL for grinding and 2 mL for rinsing. The homogenate was transferred into two 2 mL centrifuge tubes and centrifuged at 10,000 × *g* for 15 min at 4°C. The resulting supernatant (crude enzyme extract) was stored at 4°C for subsequent analysis. Four test tubes (5 mL) were prepared: two sample tubes, one control tube, and one blank tube. Each tube received the following reagents sequentially: 1.5 mL (0.05 mol/L phosphate buffer), 0.3 mL (130 mmol/L methionine solution), 0.3 mL (750 μmol/L nitroblue tetrazolium solution), 0.3 mL (100 μmol/L EDTA-2Na solution), 0.3 mL (20 μmol/L riboflavin), 0.05 mL (enzyme extract; replaced with phosphate buffer in the control tubes), and 0.25 mL distilled water. Following the reaction, the tubes were covered with black cloth to terminate the reaction. The absorbance of each tube was measured at 560 nm using the light-shielded control tube as the blank, and SOD activity was calculated.

#### 2.4.2 Peroxidase activity

Samples (0.1 g) were homogenized with 3 mL of 100 mmol/L phosphate buffer. The mortar was rinsed with 5 mL of phosphate buffer, and the combined homogenate was brought to a final volume of 10 mL and centrifuged at 10,000 × *g* for 15 min. Two test tubes were prepared: one tube contained 3 mL reaction mixture and 1 mL phosphate buffer (used as the blank control), while the other tube contained 3 mL reaction mixture and 1 mL enzyme extract. Absorbance was measured at 470 nm at 1-minute intervals.

#### 2.4.3 Catalase activity

Samples (0.1 g) were homogenized with 0.5 mL of ice-cold phosphate buffer (pH 7.0). The homogenate was transferred to a 5 mL volumetric flask, the mortar was rinsed several times with buffer, and the volume was adjusted to the mark. After thorough mixing, the solution was allowed to stand for 10 min in a refrigerator at 5°C. The supernatant was collected by centrifugation at 10,000 × *g* for 15 min. Subsequently, 0.2 mL of the extract was mixed with 1.5 mL phosphate buffer (pH 7.8) and 1 mL distilled water (the control tube received boiled enzyme extract instead). Tubes were pre-incubated in a 25°C water bath for 3 min. Then, 0.3 mL of 0.1 mol/L H_2_O_2_ was added to each tube sequentially, and timing commenced immediately. Absorbance was measured at 240 nm at 1-minute intervals.

#### 2.4.4 Malondialdehyde content

Samples (0.5 g) were homogenized in 5 mL of 5% (w/v) trichloroacetic acid (TCA) and centrifuged at 10,000 × *g* for 10 min. A 2 mL aliquot of the supernatant was mixed with 2 mL of 0.67% (w/v) thiobarbituric acid (TBA). The mixture was heated in a boiling water bath for 15 min, cooled, centrifuged, and the absorbance of the final supernatant was measured at 450 nm, 532 nm, and 600 nm.

#### 2.4.5 Soluble protein content

Samples (0.5 g) were homogenized in 5 mL of extraction buffer and centrifuged at 10,000 × *g* for 10 min. The supernatant was collected for analysis. A 1.0 mL aliquot of the supernatant was mixed with 5 mL Coomassie Brilliant Blue reagent in a test tube. After thorough shaking and incubation for 10 min, absorbance was measured at 595 nm.

### 2.5 Data statistics

Data were analyzed in Office 2021 (Microsoft Corporation, Redmond, WA, USA). Heatmaps, upset plots and Venn diagrams were created using TBtools-II v2.154 (Chen et al., 2023). One-way analysis of variance was conducted using Duncan’s method in IBM SPSS Statistics 25. Histograms were generated using Origin 2021 (OriginLab Corporation, Northampton, MA, USA). Principal Component Analysis (PCA) visualization was performed using R version 3.5.1, metabolite classification circular plots, correlation analysis and K-means analysis were generated using R version 4.2.0, differential lipid metabolite classification scatter plots were created using R version 4.2.2.

## 3 Results

### 3.1 Overview of detected lipid metabolites

**Figure 1:**
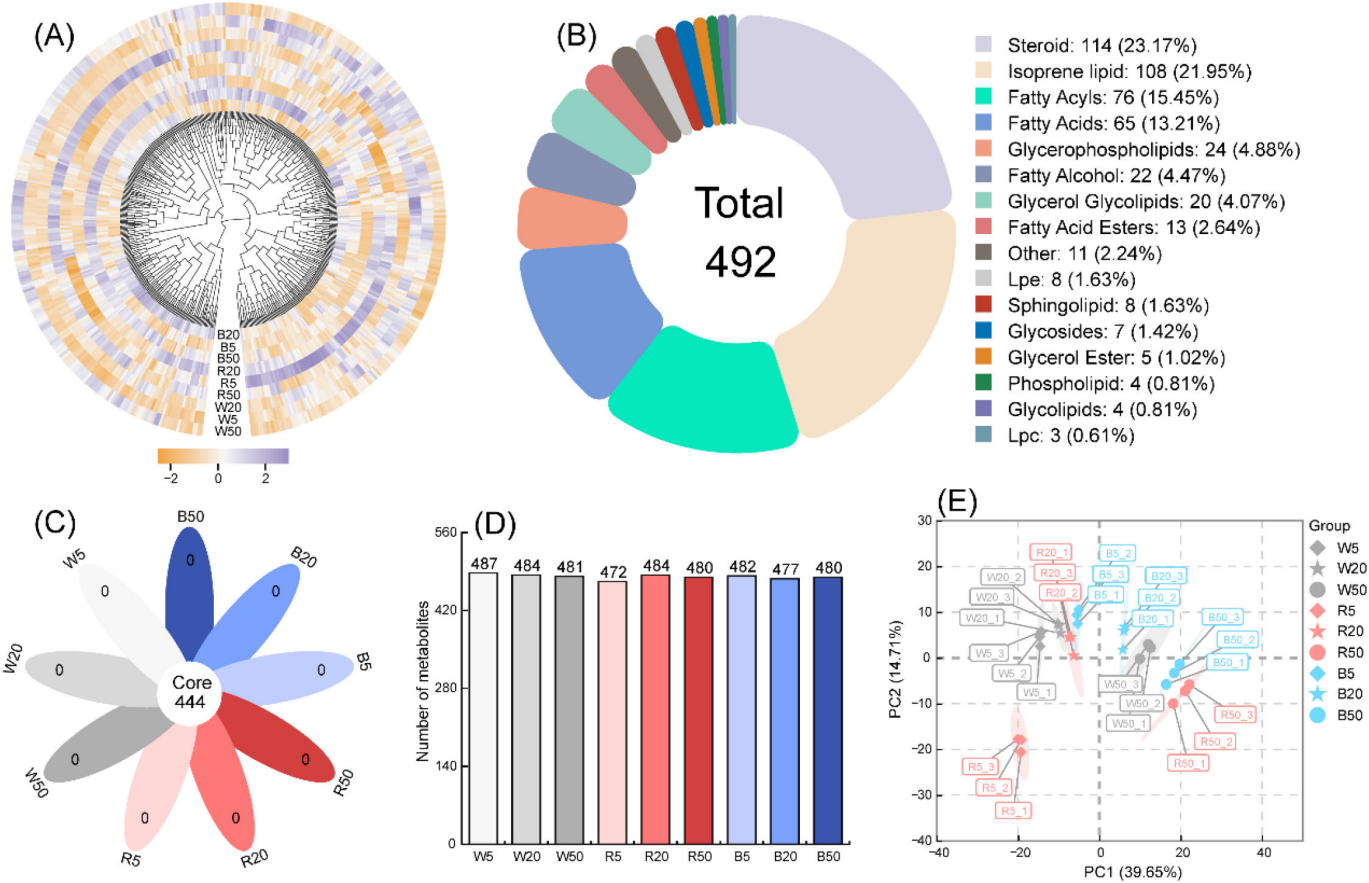
Analysis of detected lipid metabolites under different light quality and intensity treatments. **(A)** Heatmap based on the detected lipid metabolites. **(B)** Classification analysis of lipid metabolites. **(C)** Petal-style Venn diagram of detected lipid metabolites. **(D)** Distribution of the number of detected lipid metabolites per treatment. **(E)** Principal component analysis (PCA) based on the detected lipid metabolites.

In *Begonia* ‘Black Velvet’ subjected to different light qualities and intensities, we detected and annotated a total of 492 lipid metabolites. A heatmap illustrates their accumulation patterns across the treatments (**Figure 1A**). Classification analysis revealed diverse lipid types, categorized into 16 distinct classes (**Figure 1B**). Steroid and isoprene lipids were the most abundant classes, comprising 114 and 108 metabolites, respectively, each exceeding 20% of the total. Fatty acyls and Fatty acids followed, each accounting for approximately 15% of the total metabolites. Nine classes glycerophospholipids, fatty alcohol, glycerol glycolipids, fatty acid esters, other, Lpe, sphingolipid, glycosides and glycerol ester collectively contained metabolites representing 1% to 5% of the total. Conversely, metabolites classified into glycolipids, phospholipids, and Lpc (Lysophosphatidylcholines) were relatively scarce, each constituting less than 1% of the total.

A petal-style Venn diagram (**Figure 1C**) showed that 444 lipid metabolites were common to all nine treatments, with no treatment-specific metabolites detected. Furthermore, analysis of the number of lipid metabolites detected per treatment (**Figure 1D**) revealed relatively minor differences; the lowest count was 472 (R5 treatment), while the highest was 487 (W5 treatment). The plot of Principal Component Analysis (PCA) shows that triplicate samples for each treatment clustered tightly together, and the nine treatments formed distinct clusters (**Figure 1E**). PCA analysis accounted for 54.36% of the total variances, therein, PC1 explained39.65%, 14.71% for PC2 (**Figure 1E**).

### 3.2 Comprehensive analysis of differential lipid metabolites under different light quality and intensity treatments

**Figure 2:**
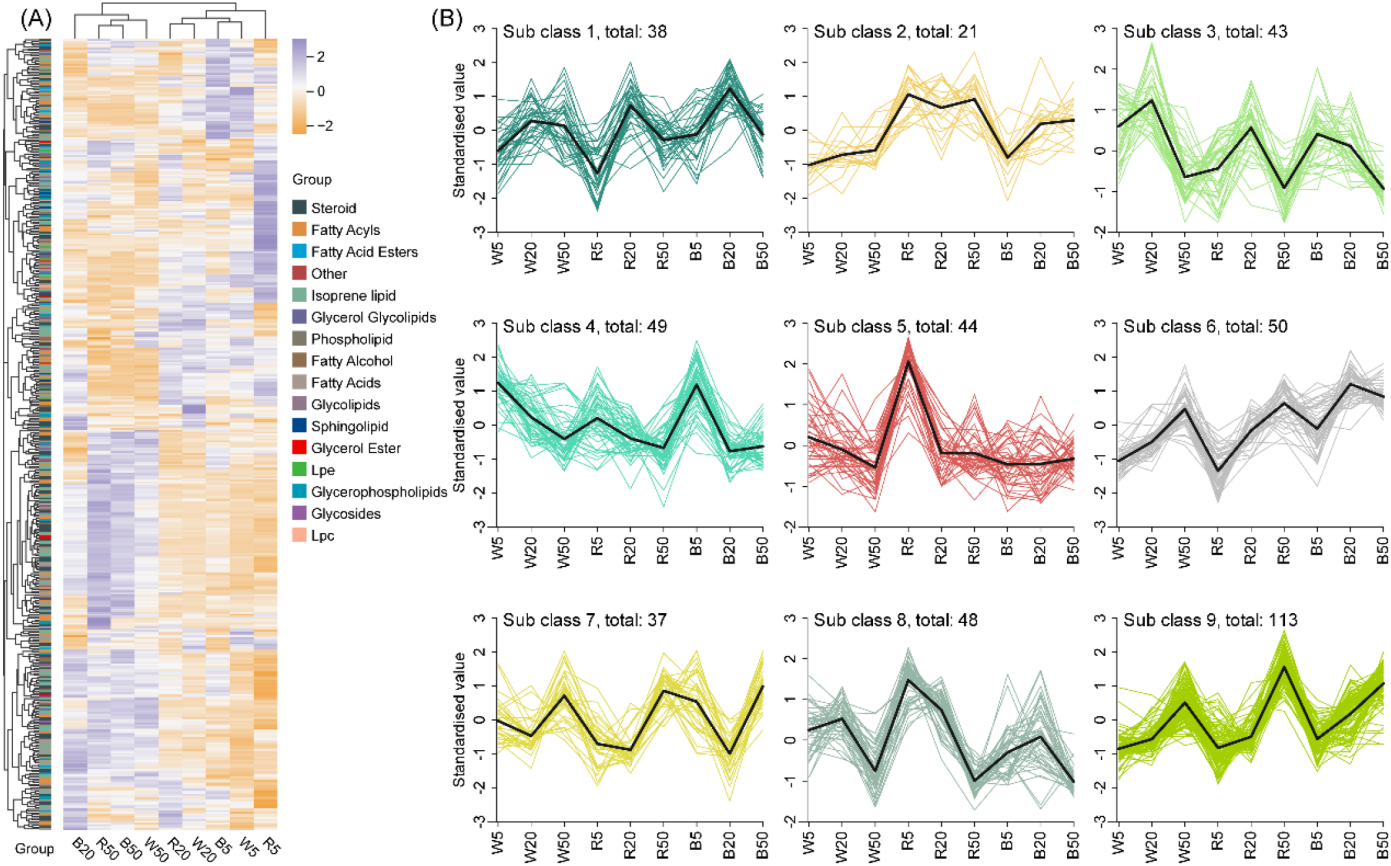
Overview of differential lipid metabolites under different light quality and intensity treatments. **(A)** Heatmap showing the relative abundance (Z-score normalized) of 443 detected DLMs across nine treatment combinations. **(B)** K-means clustering analysis partitioned the 443 DLMs into 9 distinct subclasses (Sub1-Sub9) based on their accumulation patterns across treatments.

Applying the defined differential lipid metabolites (DLMs) screening criteria, we detected and annotated 443 DLMs spanning all 16 classification classes. Moreover, the low-intensity treatments (R5, W5, B5) and the high-intensity treatments (W50, B50, R50) were separately clustered into different groups (**Figure 2A**).

To further characterize accumulation patterns, K-means clustering was performed on the 443 DLMs, partitioning them into nine distinct subclasses (**Figure 2B**). The subclasses exhibited considerable variation in size. Subclass 9 was the largest, containing 113 DLMs with high accumulation under W50, R50, and B50. In contrast, subclass 8 comprised 48 DLMs, showing the opposite pattern with low accumulation under W50, R50, and B50. Subclass 6 comprised 50 metabolites characterized by low accumulation under W5, R5, and B5, while subclass 4 contained 49 metabolites with high accumulation under these same low-intensity treatments (W5, R5, B5). Subclass 2 was the smallest, with only 21metabolites that displayed low accumulation across all white light intensities but high accumulation across all three red light intensities.

### 3.3 Analysis of differential lipid metabolites under different light quality treatments

**Figure 3:**
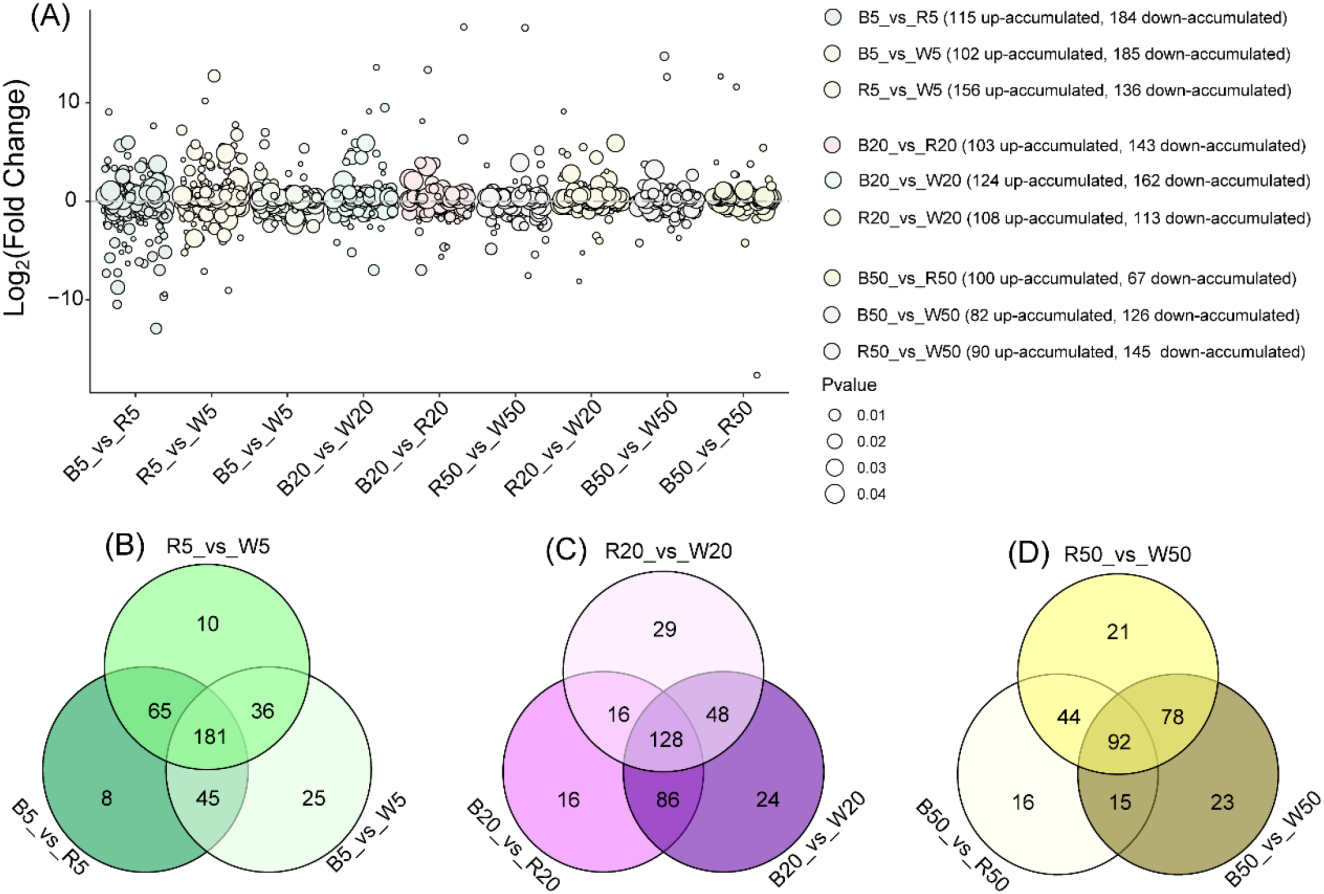
Analysis of differential lipid metabolites between different light qualities at the same intensity. **(A)** Bubble plot of differential lipid metabolites across light qualities. **(B)** Venn diagram of differential lipid metabolites in comparison groups between different light qualities at 5 μmol·m^-2^·s^-1^ intensity. **(C)** Venn diagram of differential lipid metabolites in comparison groups between different light qualities at 20 μmol·m^-2^·s^-1^ intensity. **(D)** Venn diagram of differential lipid metabolites in comparison groups between different light qualities at 50 μmol·m^-2^·s^-1^ intensity.

To assess the specific effects of light quality, we detected and annotated differential lipid metabolites (DLMs) between pairs of light qualities (blue vs red, blue vs white, red vs white) at each fixed intensity (5, 20, and 50 μmol·m^-2^·s^-1^). Bubble plots (**Figure 3A**) illustrate the number of DLMs for each comparison.

Under extremely low light intensity (5 μmol·m^-2^·s^-1^), about 290 DLMs were recorded from each three pairwise comparisons. Moreover, treatment B5 showed higher proportion of DLM accumulation (**Figure 3A**). Among the recorded DLMs, 181 DLMs were shared by these three different comparison groups (**Figure 3B**).

At 20 μmol·m^-2^·s^-1^, a higher proportion of DLM accumulation was also found under blue-light-treated samples compared to other comparison groups (**Figure 3A**). For instance, 246 DLMs for B20_vs_R20 comparison group, and 286 and 221 for B20_vs_W20 and R20_vs_W20, respectively (**Figure 3A**). Among these DLMs, 128 DLMs were shared by all comparison groups (**Figure 3C**).

Under 50 μmol·m^-2^·s^-1^ light intensity, treatment R50 showed high proportion of DLMs among these three light intensity-treated groups. Moreover, the DLM numbers were 167, 208 and 235 for comparison groups B50_vs_R50, B50_vs_W50 and R50_vs_W50, respectively (**Figure 3A**). Therein, only 92 DLMs were shared by these three comparison groups (**Figure 3D**). In addition, 25 candidate DLMs (**Supplementary File 2 Table 2**) that exhibit consistent differential accumulation among different light−quality treatments were selected.

### 3.4 Analysis of differential lipid metabolites under different light intensities treatments

**Figure 4:**
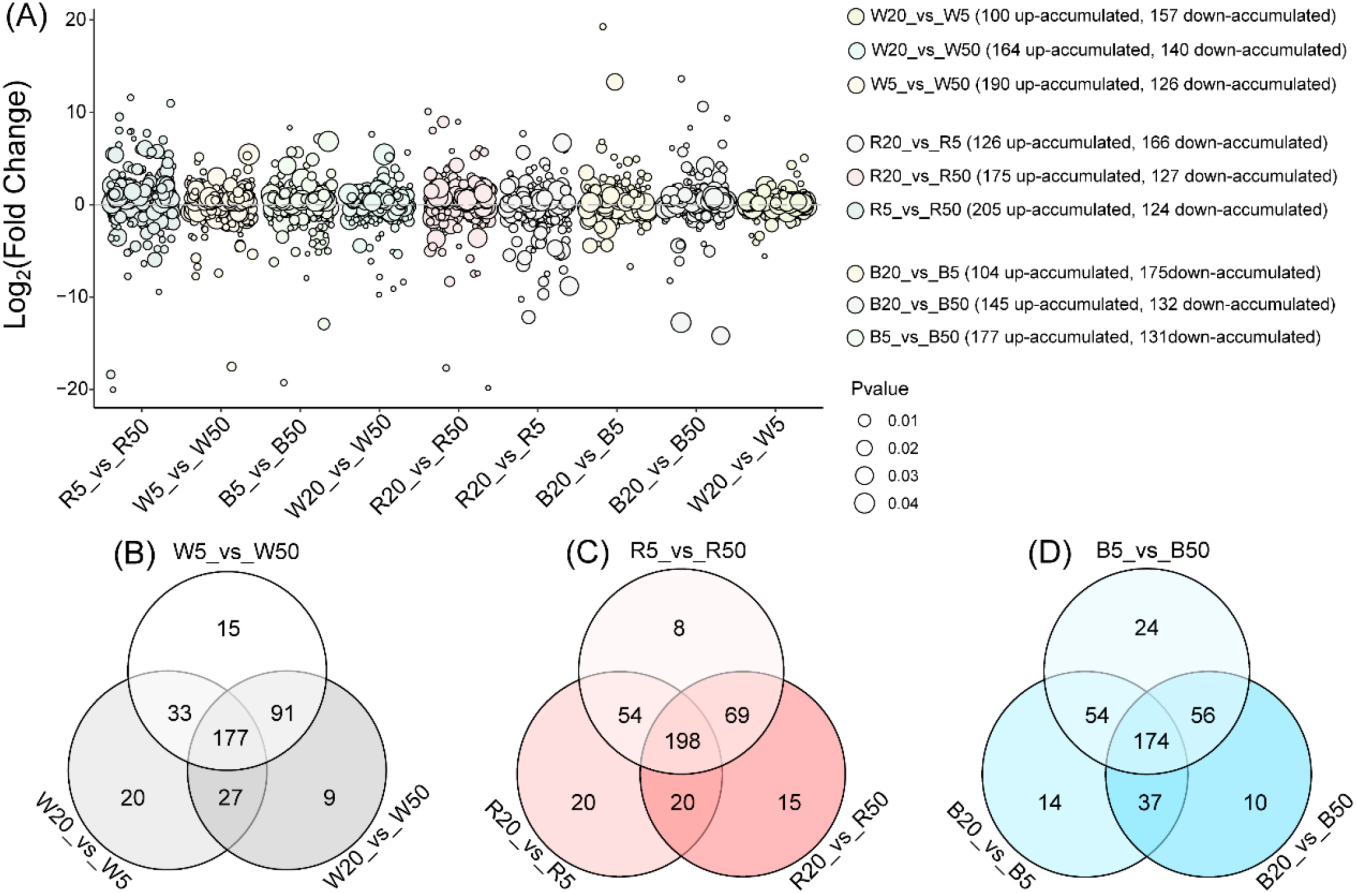
Analysis of differential lipid metabolites between different light intensities under the same light quality. **(A)** Bubble plot of differential lipid metabolites across light intensities. **(B)** Venn diagram of differential lipid metabolites in comparison groups between different light intensities under white light. **(C)** Venn diagram of differential lipid metabolites in comparison groups between different light intensities under red light. **(D)** Venn diagram of differential lipid metabolites in comparison groups between different light intensities under blue light.

To evaluate the effects of light intensity, we detected and annotated differential lipid metabolites (DLMs) between pairs of intensities (5, 20, and 50 μmol·m^-2^·s^-1^) within each light quality (white, red, and blue). Bubble plots summarize the number of DLMs for each pairwise comparison (**Figure 4A**).

Under white light, high-light-treated samples (W50, W20) showed higher proportion of DLMs accumulation compared to W5 treatment (**Figure 4A**). Moreover, treatment W50 has the highest metabolite accumulation under white light quality (**Figure 4A**). And 257, 304, and 316 DLMs were separately found from comparison groups W20_vs_W5, W20_vs_W50, and W5_vs_W50. Therein, 177 DLMs were shared by these three different light intensity-treated comparison groups (**Figure 4B**).

Under red-light-treated samples, the comparisons R20_vs_R5, R20_vs_R50, and R5_vs_R50 contained 292, 302, and 329 DLMs, respectively. Treatment R50 has higher accumulation of DLMs compared to other samples under low light intensity (**Figure 4A**). Moreover, a total of 198 DLMs were shared among all three red-light-treated comparisons (**Figure 4C**).

Under blue-light-treated samples, treatment B50 showed the higher DLMs accumulation compared to other comparisons (**Figure 4A**). The comparison groups B20_vs_B5, B20_vs_B50, and B5_vs_B50 separately annotated 279, 277, and 308 DLMs. Moreover, 174 common DLMs were shared by these three different comparison groups (**Figure 4D**). In addition, 23 candidate DLMs, showing consistent differential accumulation across intensity gradients were finally selected through integrated analysis among white, red, and blue light comparison groups.

### 3.5 Interactive effects of light quality and light intensity on lipid metabolites

**Figure 5:**
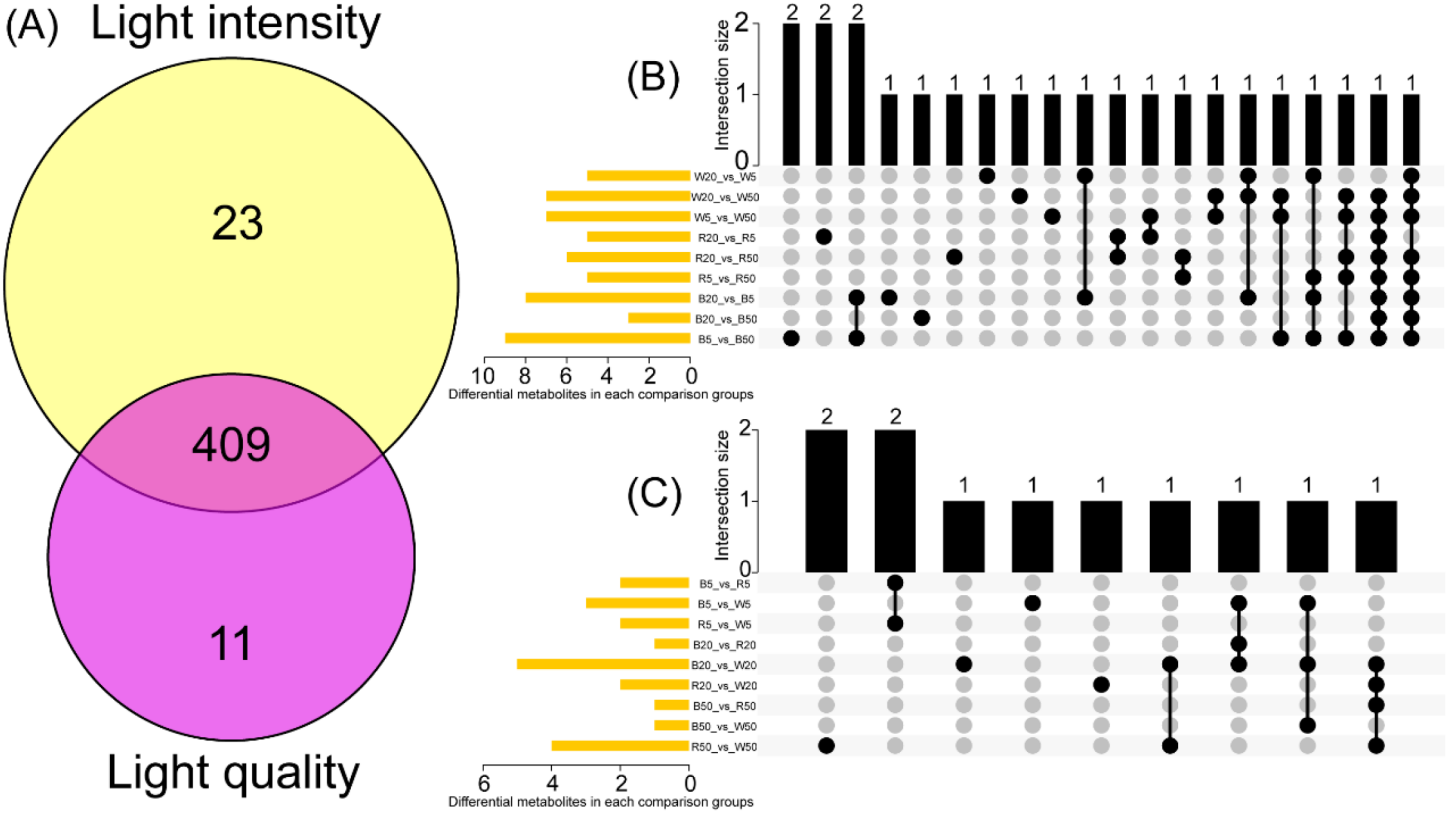
Combined effects of light quality and intensity on lipid metabolites. **(A)** Venn diagram of lipid metabolites under different light qualities and intensities. **(B)** UpSet plot of light intensity-specific differential lipid metabolites. **(C)** UpSet plot of light quality-specific differential lipid metabolites.

Among these 432 DLMs, 420 DLMs were recorded under different light quality-treated samples, 432 for light intensity-treated samples (**Figure 5A**). Therein, 409 metabolites overlapped (**Figure 5A**), indicating a shared response to both factors. Moreover, certain intensity-specific DLMs (e.g., POS_t1278, POS_t961) showed differential accumulation under multiple light qualities (**Figure 5B**). Likewise, some quality-specific DLMs (e.g., POS_t569) responded differentially across all three intensities under particular spectral conditions (**Figure 5C**).

### 3.6 Comprehensive physiological profiling across light intensities and qualities

**Figure 6:**
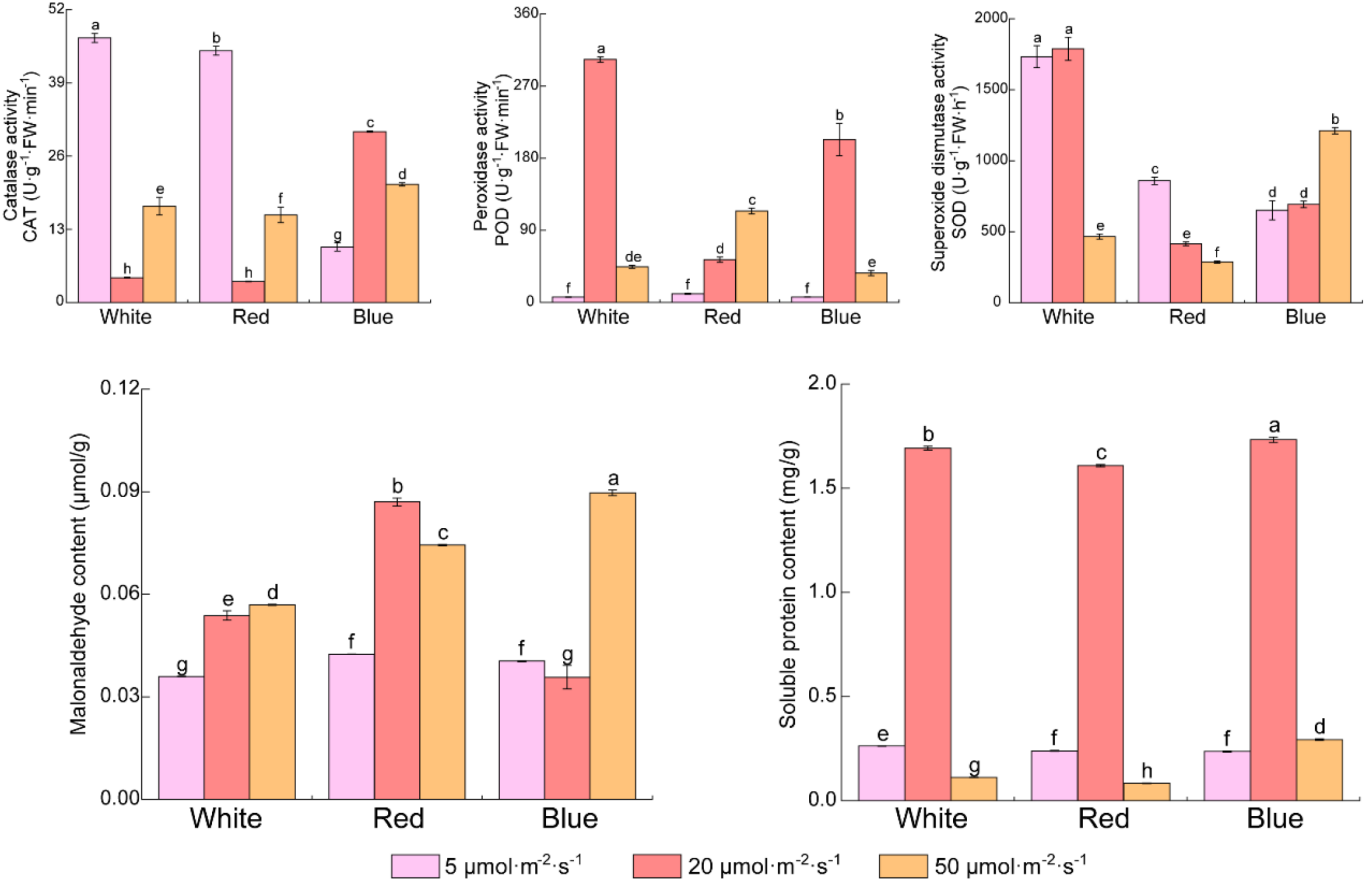
Effects of light quality and intensity on physiological profiles in *Begonia* ‘Black Velvet’. Values are presented as mean ± standard error (SE) of three biological replicates (n=3). Different lowercase letters above the bars within each panel indicate statistically significant differences among treatments for that specific physiological index (one-way ANOVA followed by Duncan’s test, *p* < 0.05).

The activities of antioxidant enzymes (SOD, POD, CAT), malondialdehyde (MDA) content, and soluble protein (SP) contents varied across light treatments (**Figure 6**). Under 5 μmol·m^-2^·s^-1^, white- and red-light-treated samples showed the highest CAT activity, while SOD activity was lowest under blue light. In contrast, MDA contents were low across all qualities at this intensity. At optimal intensity (20 μmol·m^-2^·s^-1^), white light elicited peak synergistic activation of POD and SOD (303.33 U POD, 1788.24 U SOD, respectively). Soluble protein content reached maximal levels across all light treatment (1.61–1.73 mg/g), while blue light achieved minimal MDA accumulation (0.036 μmol/g). Under high-stress (50 μmol·m^-2^·s^-1^) conditions, divergent antioxidant strategies emerged. Blue light triggered an SOD surge (1211.6 U, + 75% vs 20 μmol) with concurrent MDA escalation (0.090 μmol/g, +151%). Red light exhibited POD compensation (114.11 U, doubling 20 μmol levels) despite SOD collapse (decreased 31%). Conversely, white light suffered catastrophic enzyme suppression (POD decreased 85%, SOD decreased 74%) alongside soluble protein depletion (decreased 93%). Intriguingly, blue light uniquely maintained elevated soluble protein (0.29 mg/g, +24%) and conserved CAT activity (21.0 U) contrasting with 64–65% CAT suppression under white and red light conditions.

### 3.7 Integrated lipid metabolite-physiology networks

**Figure 7:**
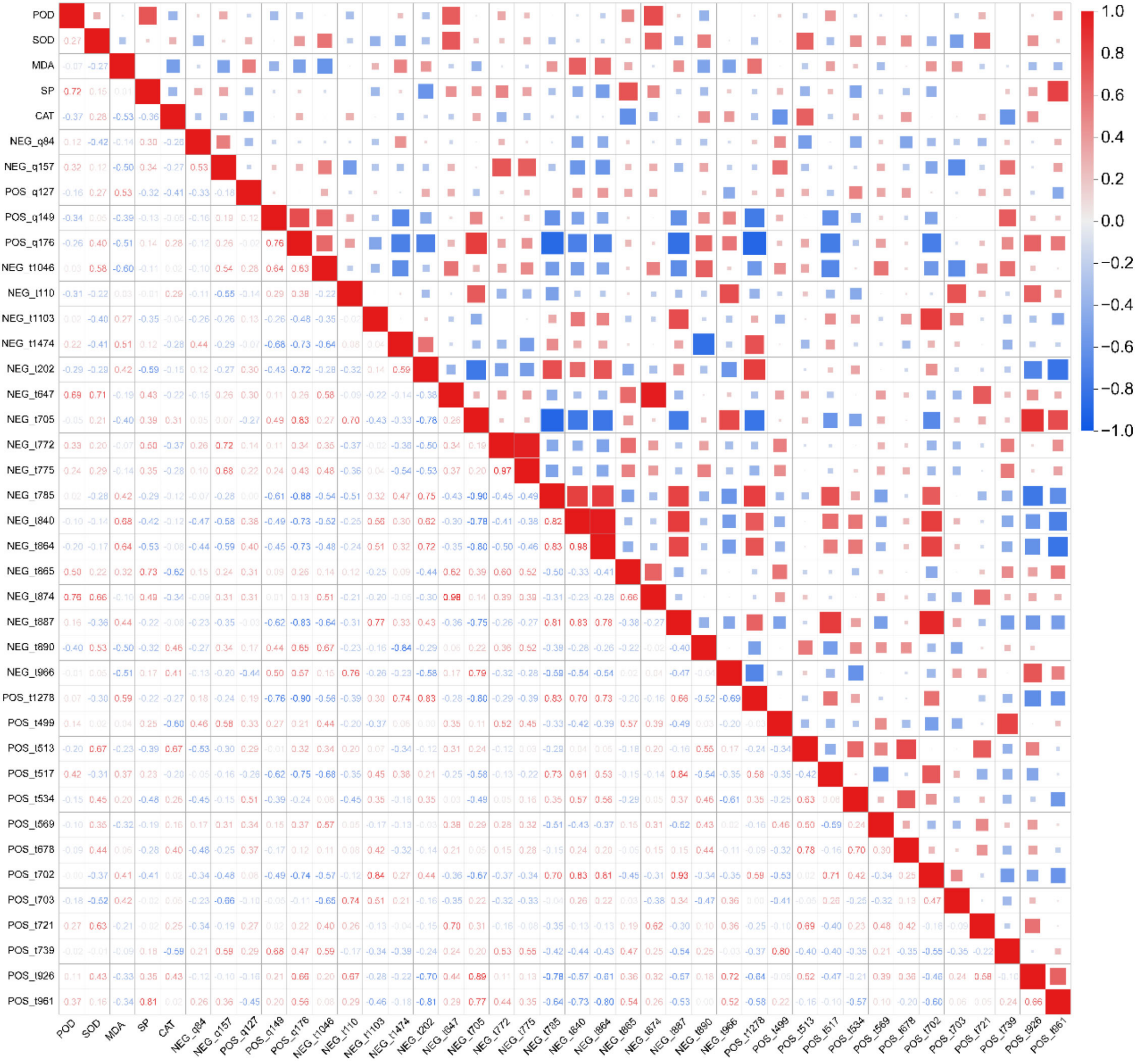
Correlation network between candidate lipid metabolites and physiological indices. The heatmap illustrates the Pearson correlation coefficients (*r*) between the accumulation levels of 35 key lipid metabolites and five physiological indicators. Color gradient and square size jointly represent the strength and direction of correlation: Large red squares indicate strong positive correlations (*r* approaching +1), large blue squares indicate strong negative correlations (*r* approaching -1), and small squares indicate weak correlations (*r* approaching 0).

Correlation analysis integrating physiological indices and candidate lipid metabolites revealed several significant associations (**Figure 7**). Two lipid metabolites, NEG_t647 and NEG_t874 exhibited significant positive correlations with POD (correlation coefficient > 0.6; *p* < 0.001). Four lipid metabolites, NEG_t647, NEG_t874, POS_t513, and POS_t721, showed similarly strong positive correlations with SOD (correlation coefficient > 0.6; *p* < 0.001). Two lipid metabolites, NEG_t840 and NEG_t864, were significantly positively correlated with MDA (*p* < 0.001), while NEG_t865 and POS_t961correlated positively with SP (*p* < 0.001). Additionally, NEG_t865, POS_t499, and POS_t513, demonstrated significant positive correlations with CAT (*p* < 0.001).

Overall, nine lipid metabolites, including NEG_t647, NEG_t840, NEG_t864, NEG_t865, NEG_t874, POS_t499, POS_t513, POS_t721, and POS_t961 showed significant correlations (*p* < 0.001) with physiological indices. Among these, three were previously identified as responsive to both light quality and intensity, four were primarily responsive to intensity, and two were primarily responsive to quality.

### 3.8 Transcriptome sequencing and quality control

Total RNA from all samples was subjected to paired-end transcriptome sequencing. Raw sequencing data were processed to obtain clean data by removing adapters and low-quality reads. Quality metrics are summarized in **Supplementary File 4 Table S4**). Clean reads per sample ranged from 19.64 million to 30.44 million, corresponding to a total base count between 5.83 Gb and 8.93 Gb. GC content was consistent across samples (44.68% to 47.00%). Q30 scores were all above 95.82%, with most samples exceeding 96% and several reaching over 99%, indicating high base-calling accuracy and reliable sequencing quality. These metrics confirm the sequencing data are of high quality and suitable for downstream transcriptome assembly and differential expression analysis.

### 3.9 Transcriptomic analysis of key lipid metabolic pathways

To elucidate the transcriptional basis of light-induced lipid remodeling, we performed RNA sequencing across all nine treatment groups (three light qualities × three intensities). We focused on two core biosynthesis pathways, including steroid and fatty acid metabolism that were prominently represented among the candidate lipid metabolites.

**Figure 8:**
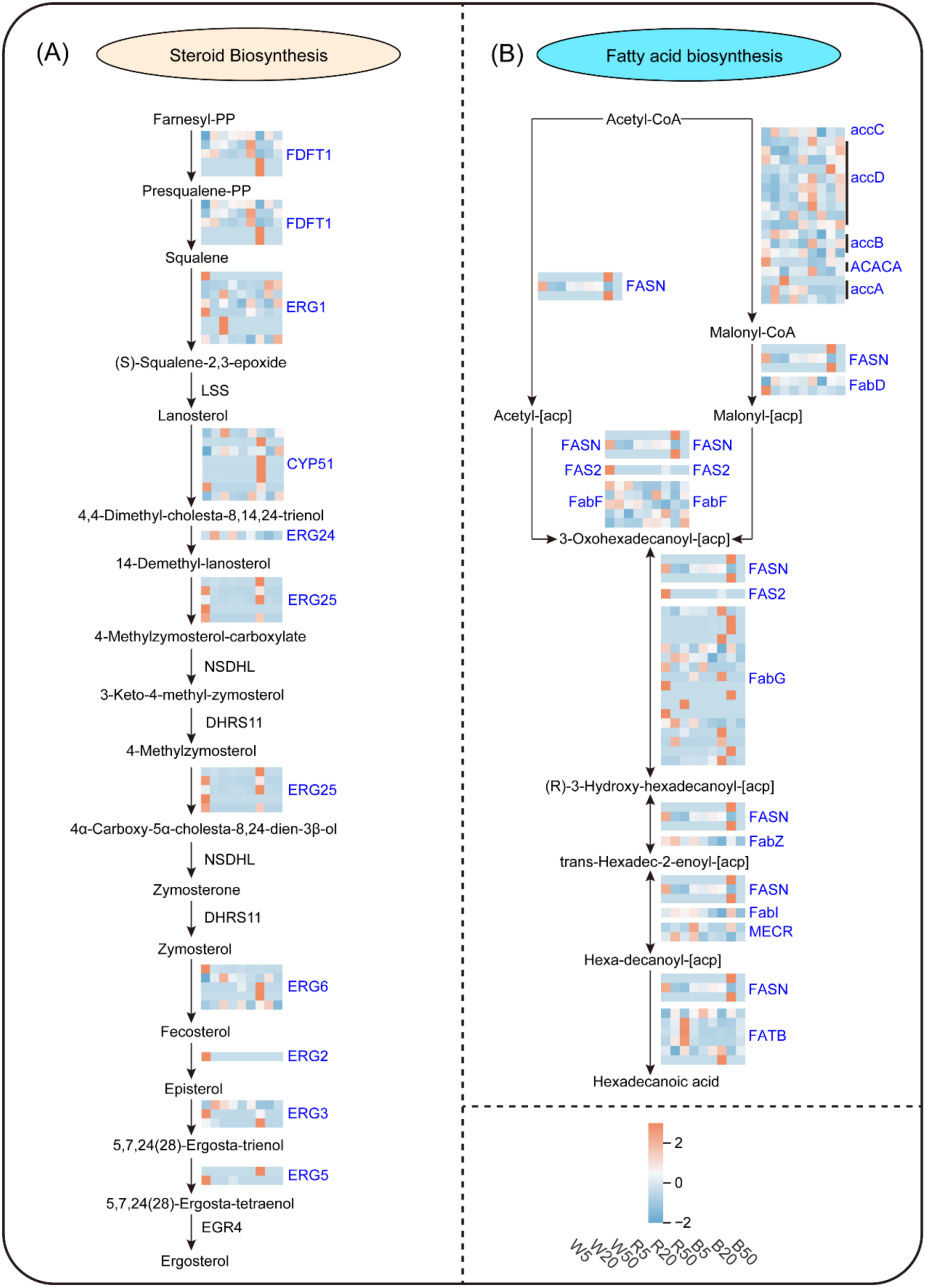
Metabolic pathway analysis of candidate lipid metabolites in *Begonia* under different light qualities and intensities. **(A)** Steroid biosynthesis. **(B)** Fatty acid biosynthesis. Gene nodes are colored according to their relative abundance (Z-score) across treatments (color scale: high in orange/red, low in blue). Gene symbols in blue indicate differentially expressed genes (DEGs).

Steroid biosynthesis pathway. We mapped 38 DEGs onto nine enzymatic steps leading to ergosterol (**Figure 8A**). Seven *ERG*-family genes, including *ERG1*, *ERG3*, *ERG5*, *ERG6*, *ERG24*, and *ERG25*, exhibited expression levels significantly correlated (r > 0.6, *p* < 0.001) with the accumulation of key steroid metabolites (**Figure 8A**; **Supplementary File 5 Table S5**).

Fatty acid biosynthesis pathway. Similarly, we identified 57 DEGs across all steps of the fatty acid biosynthesis pathway from acetyl-CoA to hexadecanoic acid (**Figure 8B**; **Supplementary File 5 Table 5**.). Nine of these DEGs, including four *accD*, two *accB*, two *FabF*, and one *FATB*, exhibited strong correlations (r > 0.6, *p* < 0.001) with fatty-acyl metabolite levels (**Figure 8B**).

The coordinated expression of these pathway-specific DEGs, particularly the *ERG* family genes for steroid synthesis and *accD*/*FabF* for fatty acid metabolism, provides a transcriptional framework that supports the observed light-dependent lipid compositional changes.

## 4 Discussion

### 4.1 Overall lipid metabolic response patterns to light environment

This study systematically deciphers how light quality and intensity modulate lipid metabolism in the shade-adapted *Begonia* ‘Black Velvet’. We detected and annotated 492 lipid metabolites across nine light treatments, classified into 16 categories with steroid (23.17%) and isoprene lipids (21.95%) predominating. Principal component analysis (PCA) revealed that light intensity was more strongly associated with the overall differences in light quality on global lipid profiles, as treatments clustered primarily by intensity (5/20/50 μmol·m^-2^·s^-1^) and secondarily by spectral quality. The high degree of overlap (444 out of 492) in metabolites across all treatments indicates a stable core lipidome. The absence of treatment-unique metabolites and the variation in metabolites detected per treatment (472–487) together imply that light regimes primarily induce quantitative remodeling of a shared metabolite set, rather than a wholesale restructuring. Notably, despite this high commonality, treatments grouped more strongly by intensity in the PCA, underscoring the dominant role of light intensity in driving these quantitative changes.

### 4.2 Spectral- and intensity-specific regulation of lipid metabolism

Specific wavelengths modulate lipid metabolism through dedicated photoreceptors (Lim et al., 2023). In this study on *Begonia*, we annotated 25 differential lipid metabolites (DLMs) influenced primarily by light quality, including steroids and fatty acyls. Notably, pregnanetriol accumulation was specifically enhanced under blue light. This aligns with known blue-light responses in other species, where cryptochrome signaling upregulates fatty acid desaturase genes, increasing polyunsaturated fatty acids (PUFAs) that can contribute to membrane adaptation (Ma et al., 2016).

Concurrently, light intensity exerted a dominant quantitative effect. We annotated 23 DLMs significantly influenced by intensity, with metabolites such as (2R,3R,4S,5S,6R)-2-[(2R)-2,3-Dihydroxypropoxy]-6-(hydroxymethyl)oxane-3,4,5-triol peaking at 20 μmol·m^-2^·s^-1^, coinciding with maximal soluble protein levels. This suggests an optimal intensity for biosynthesis. Higher intensity (50 μmol·m^-2^·s^-1^) broadly increased the abundance of numerous lipids, a shift consistent with a redirection of carbon flux under high light stress (Alboresi et al., 2016; Ge et al., 2025).

Critically, we found extensive synergistic regulation: 409 lipid metabolites responded to both factors. For example, low-intensity blue light (B5) elevated putative PUFA-rich membrane lipids, while high-intensity blue light (B50) shifted accumulation toward potential antioxidant species like chabrolosteroid E. These coordinated changes underscore that lipid remodeling in *Begonia* is fine-tuned by the interplay of spectral signal and energy availability (photon flux).

It is important to note that while our lipidomic profiling revealed significant remodeling of lipid classes including potential PUFA-containing species, direct quantitative measures of membrane fluidity parameters (e.g., galactolipid ratios or fatty acid unsaturation indices) were not within the scope of this widely-targeted study. Therefore, the proposed link between observed lipid changes and membrane fluidity adjustment remains a plausible hypothesis based on established knowledge of lipid function and the stress-marker correlations observed here. Future targeted lipid analyses and biophysical assays will be valuable to directly test this mechanism in *Begonia*.

### 4.3 Functional roles of lipid remodeling in energy storage and membrane stabilization

Lipids are pivotal for plant adaptation to light, serving dual roles in energy storage and membrane integrity (Burgos et al., 2011). Our data reveal that *Begonia* ‘Black Velvet’ modulates these functions differentially in response to light intensity and quality.

Energy storage is optimized under moderate, non-stressful light. The significant increase in glyceroglycolipids at 20 μmol·m^-2^·s^-1^, particularly under white light, aligns with peak soluble protein levels, indicating a metabolic state favoring biosynthesis. This light regime provides sufficient photosynthate to activate fatty acid synthesis and storage lipid assembly (Kowalczyk et al., 2021). These reserves can be mobilized via β-oxidation to sustain metabolism under subsequent stress (Zheng et al., 2016).

Membrane stabilization involves dynamic adjustments in lipid composition. Under high light (50 μmol·m^-2^·s^-1^), we observed an increased relative abundance of lipid species that are typically enriched in polyunsaturated fatty acids (PUFAs). In many plants, such a shift is a known adaptive mechanism that could help maintain membrane fluidity and mitigate photo-oxidative damage (Carmona-Salazar et al., 2015; Wu et al., 2023). Concurrently, specific glycerophospholipids like phosphatidylcholine (PC) underwent compensatory changes, which can enhance membrane stability and protein function (Wang et al., 2020; Morales-Martínez et al., 2022). These putative adaptive adjustments are reflected in the correlation of key lipids (e.g., NEG_t840, NEG_t864) with oxidative stress markers (MDA), and others (e.g., NEG_t865, POS_t513) with antioxidant enzymes (CAT/SOD).

The physiological profiling revealed that soluble protein content uniquely peaked at 20 μmol·m^-2^·s^-1^ across light qualities, a trend distinct from antioxidant enzymes and MDA. This indicates that 20 μmol·m^-2^·s^-1^ represents a light optimum for growth in this shade-adapted species, where resources are allocated to protein synthesis and general metabolism. In contrast, at 50 μmol·m^-2^·s^-1^, the activation of specific antioxidant defenses (SOD, POD) and the rise in MDA signify a shift in priority from growth to stress acclimation. The decline in soluble protein under high light likely reflects resource reallocation toward stress-responsive proteins and/or protein degradation under oxidative pressure. Thus, the soluble protein peak serves as a biomarker for favorable conditions, while antioxidant and MDA responses mark the onset of light stress, explaining their divergent patterns (Dahl et al., 2015; Song et al., 2016).

As an understory-adapted species, *Begonia*’s lipid plasticity, characterized by PUFA enrichment and sterol-mediated membrane reinforcement contrasts with sun-tolerant models. Our findings suggest precision horticulture strategies: blue-enriched spectra at ≤ 20 μmol·m^-2^·s^-1^ can enhance antioxidant capacity, while intensity gradients can be tuned to optimize energy storage (20 μmol) or stress resilience (50 μmol).

### 4.4 Differential regulatory logic underlying the accumulation of specific lipid classes

Nine key lipids, including chabrolosteroid E (NEG_t647) (regulated by both light intensity and quality) and culobophylin C (POS_t721) (quality-specific) emerged as central to photoprotection. The distinct response patterns of key lipid metabolites to light intensity versus quality reveal fundamental differences in their biosynthetic regulation. The contrasting responses of two key photoprotective lipids, POS_t721 (culobophyllin C, an isoprene lipid) and NEG_t647 (chabrolosteroid E, a steroid) highlight distinct biosynthetic triggers. POS_t721 accumulated primarily under specific light qualities (e.g., blue light) with little change across intensities, suggesting its biosynthesis is directly coupled to photoreceptor signaling (e.g., cryptochrome activation of the plastidial MEP pathway) (Vranová et al., 2013). In contrast, NEG_t647 responded synergistically to both light quality and intensity, reflecting a homeostatic adjustment to membrane stress. Steroids, as key membrane components, likely accumulate under high light to stabilize membranes against photo-oxidative damage, a need that scales with photon flux (Ferrer et al., 2017). Thus, *Begonia* employs a layered regulatory strategy: light quality fine-tunes specific signaling molecules (e.g., isoprene lipids), while light intensity broadly recalibrates structural components (e.g., steroids) in response to cellular stress.

### 4.5 Transcriptional regulatory mechanisms underlying light-induced lipid changes

Light quality regulates gene expression through photoreceptor-specific signaling pathways (Sanchez et al., 2020), ultimately affecting lipid synthesis (Poitelon et al., 2020). Our transcriptomic data show that key lipid metabolic genes (e.g., *accD*, *FabF*, and *ERG* family members) are regulated by light. This aligns with the established paradigm where photoreceptors (e.g., phytochromes for red light, cryptochromes for blue light) relay signals through intermediates like the COP1/SPA complex and PIF transcription factors to reshape transcription (Llorente et al., 2017; Maldini et al., 2015). The upregulation of fatty acid desaturation-related genes under blue light may be linked to the cryptochrome-PIF axis (Ma et al., 2016), while red light effects on membrane lipid genes likely involve phytochrome signaling. Although we did not directly validate upstream receptors, our data position lipid metabolic reprogramming as a key downstream response to light cues.

These transcriptional changes provide a mechanistic basis for the observed lipid remodeling. We focused on a core set of regulatory genes whose expression showed strong correlations with metabolite accumulation. Within the fatty acid pathway, *accD* (along with *accB*) controls the committed step of synthesis initiation (Madoka et al., 2002), while *FabF* regulates chain elongation and influences unsaturated fatty acid supply (Morgan-Kiss and Cronan, 2008); in *Xanthoceras sorbifolium*, *FabF* collaborates with other enzymes to promote specific fatty acid accumulation (Shi et al., 2024). All differentially expressed steroid-related genes belonged to the *ERG* gene family, central to plant steroid and lipid metabolism (Bishop and Koncz, 2002).

Future research should focus on elucidating the subfunctional divergence within the *ERG* gene family and employing synthetic biology approaches to optimize metabolic flux for enhancing stress resistance and high-value lipid production. It should be noted that the 14−day treatment period, while ecologically relevant for capturing integrated adaptive responses in this perennial shade−adapted species, may combine direct photoregulatory signals with ontogenetic adjustments during leaf development. Future time−course experiments could help disentangle the acute effects of light quality and intensity from longer−term developmental reprogramming of lipid metabolism.

## 5 Conclusion

This study elucidates the central role of light environment in driving lipid metabolic adaptation in *Begonia* ‘Black Velvet’, revealing that light intensity is the dominant factor for lipid remodeling: moderate intensity (20 μmol·m^-2^·s^-1^) promotes glyceroglycolipids for energy storage, while higher intensity (50 μmol·m^-2^·s^-1^) shifts the lipid profile toward a higher relative abundance of lipids commonly associated with unsaturation, which may represent an adaptive response to enhance membrane stability under mild stress. Spectral quality exerts distinct effects, with blue light elevating antioxidants like chabrolosteroid E and polyunsaturated fatty acids (PUFAs), and red light specifically upregulating phosphatidylglycerol to maintain photosystem II integrity. Significant synergistic regulation occurs, evidenced by 409 lipid metabolites responding to both factors; notably, low-intensity blue light (B5) enriches PUFA-rich membranes, whereas high-intensity blue light (B50) shifts lipid profiles toward protective species. Physiological integration confirms lipids as key mediators of photoprotection, linking specific metabolites (e.g., NEG_t865, POS_t513) to antioxidant enzyme activity (CAT/SOD) and oxidative damage markers (MDA). Underpinning these metabolic shifts is transcriptional control, involving *ERG* genes regulating steroid pathways and key enzymes like *accD* and *FabF* modulating fatty acid unsaturation. These mechanistic insights enable precision cultivation strategies, such as using blue-enriched spectra at ≤ 20 μmol·m^-2^·s^-1^ to boost antioxidant capacity for ornamental quality, or implementing light gradients to optimize either energy storage (20 μmol) or stress resilience (50 μmol). Future research should target ERG gene subfunctionalization and lipid metabolic engineering to enhance stress tolerance in *Begonia* and related shade-adapted species.

## Data Availability

The original contributions presented in the study are included in the article/**Supplementary Material**. Further inquiries can be directed to the corresponding authors.
